# Shifting Epicenters: The Dynamic Regional Dispersal of SARS-CoV-2 Omicron in Poland

**DOI:** 10.3390/v18050520

**Published:** 2026-04-30

**Authors:** Marcin Horecki, Karol Serwin, Miłosz Parczewski

**Affiliations:** Department of Infectious, Tropical Diseases and Immune Deficiency, Pomeranian Medical University in Szczecin, 71-455 Szczecin, Poland; karol.serwin@pum.edu.pl (K.S.); milosz.parczewski@pum.edu.pl (M.P.)

**Keywords:** SARS-CoV-2, Omicron, phylogeography, Bayesian inference, genomic epidemiology, viral dispersal, Poland, COVID-19 surveillance

## Abstract

The evolution and spatial dissemination of SARS-CoV-2 Omicron subvariants have been characterized by rapid lineage replacement and complex transmission dynamics influenced by regional connectivity. This study presents a comprehensive discrete phylogeographic analysis of 90,136 SARS-CoV-2 sequences collected in Poland from 2022 to 2024 to reconstruct the dispersal dynamics of major Omicron lineages, including BA.1, BA.2, BA.5, CH.1, XBB.1, and JN.1. Utilizing Bayesian statistical frameworks, we identified significant viral transitions between the 16 Polish voivodeships and established variant-specific dominance windows ranging from 2 to 4 months. Our findings reveal a highly dynamic epidemic landscape with shifting regional epicenters. The initial BA.1 wave was primarily driven by the Mazovian voivodeship, accounting for 36.1% of outward migration events. This pattern shifted dramatically with the rise in BA.2, which was centered in the industrial Silesian region in the south-west, a densely populated area with strong economic ties to neighboring countries, potentially reflecting a different introduction or transmission dynamic. Furthermore, the epidemic landscape continued to reconfigure during the BA.5 wave, marked by the emergence of new transmission hubs in eastern border regions such as Lublin. Subsequent lineages exhibited distinct geographic signatures: BA.5 spread broadly along the Baltic-central corridor, CH.1 was centered in the north-east, XBB.1 re-emerged in the west-central region of Greater Poland, and JN.1 was driven overwhelmingly by Lesser Poland. These transitions highlight that regional transmission hubs are transient and influenced by local factors such as population density, cross-border mobility, and socio-economic connectivity. This study underscores the critical value of dense genomic surveillance in identifying evolving dispersal routes to inform adaptive, region-specific public health interventions.

## 1. Background

The Severe Acute Respiratory Syndrome Coronavirus 2 (SARS-CoV-2) pandemic, which began in late 2019, has profoundly impacted Poland, affecting public health, the economy, and social dynamics across the nation. The first confirmed case in Poland was reported on 4 March 2020 [1], leading to a series of governmental measures aimed at curbing the virus’s spread, including lockdowns, travel restrictions and a nationwide vaccination campaign initiated in late 2020 [2].

As of February 2026, Poland has recorded almost 6.8 million confirmed Coronavirus Disease (COVID-19) cases and approximately 121,000 deaths [3], highlighting the pandemic’s significant toll on the population and the healthcare system.

Prior to the emergence of Omicron in late 2021, Poland experienced four distinct epidemic waves driven sequentially by the original SARS-CoV-2 strain, the Alpha variant, and the Delta variant, which cumulatively resulted in over four million confirmed infections and severe healthcare burdens [4,5,6].

Following the first confirmed case of Omicron BA.1 in England on 27 November 2021, its prevalence increased rapidly across all English regions and worldwide [4]. In Poland, BA.1 was first detected on 16 December 2021 and soon posed significant public health challenges [5]. Omicron, designated a variant of concern by the World Health Organization (WHO) [6] due to its high transmissibility and potential impact on existing public health measures, rapidly became the predominant strain circulating in the country, exacerbating the ongoing COVID-19 pandemic and leading to increased hospitalization rates and deaths across the nation [7].

In response to the Omicron surge, Polish health authorities implemented a series of restrictive measures [8] aimed at curbing the spread of the virus, including a flight ban from southern Africa and tightened quarantine protocols. These efforts came as Poland faced a steep rise in COVID-19 cases, particularly in March 2022, when hospitalizations surged and the nation surpassed 100,000 deaths attributed to the virus [9].

Tracking the emergence, introduction and spread of SARS-CoV-2 variants of concern is essential for informing public health strategies. To gather insights into the patterns and dynamics of virus spread, we conducted discrete phylogeographic analysis of the SARS-CoV-2 epidemic utilizing the BEAST software package v1.10.4 [10]. This method leverages Bayesian statistical frameworks to trace the spatial evolutionary history of SARS-CoV-2 in many regions, revealing complex transmission routes in countries influenced by geographic and socio-economic factors [11,12].

Investigating the spread and changing dominance of specific Omicron lineages may offer insight into cross-country divergence in variant replacement. In the Polish context, such patterns may differ from those seen in adjacent countries. One possible contributing factor is the substantial influx of war-displaced individuals from Ukraine in 2022—a population in which vaccination coverage has been considerably lower compared to other regions. Specific areas within Poland may act as regional transmission hubs characterized by increased levels of viral dissemination. However, these centers may change in response to the emergence and dominance of new Omicron variants. Our study aimed to investigate the dominance and transmission patterns of SARS-CoV-2 BA.1.1.529, BA.2.*, BA.5.*, XBB.1.*, JN.1.* and CH.1.* variants in Polish regions (i.e., first administrative level) during the Omicron phase from 2022 to 2024. By examining the movement of the virus across different regions, we identified key pathways driving transmission [13].

Insights into regional patterns of dissemination of Omicron variants provide valuable guidance for public health interventions to mitigate future outbreaks. Analyzing these dynamics may enhance our understanding of country-specific SARS-CoV-2 epidemics and inform prospective surveillance and preparedness efforts.

## 2. Methods

### 2.1. Sequence Collection

For this study, we gathered all available SARS-CoV-2 genome sequences using the GISAID database downloads section [14] (accessed on 5 September 2024). The data spanned from 2 March 2020 to September 2024, marking the end of our data collection. We focused on sequences from Poland. The initial GISAID dataset consisted of 16,906,504 sequences. We filtered sequences for Poland using the built-in Linux tool awk [15] and augur filtering tools—a part of the Nextstrain package [16]. We also specified a date range between 28 January 2020 and 30 June 2024, where sufficient data were available. Ambiguous dates were removed. Finally, we applied another part of the Nextstrain package—the python_sanitize.py script—which fixed the column names of the metadata file and parsed the location names.

As the location data in the GISAID metadata file often contained inadequate entries, we standardized the region names to match the 16 correct Polish voivodeship names. Entries without location data were removed as they could not be used in phylogeographic discrete analysis. The final number of Polish sequences with location data was 90,136 sequences.

### 2.2. Subsampling

**Figure 1:**
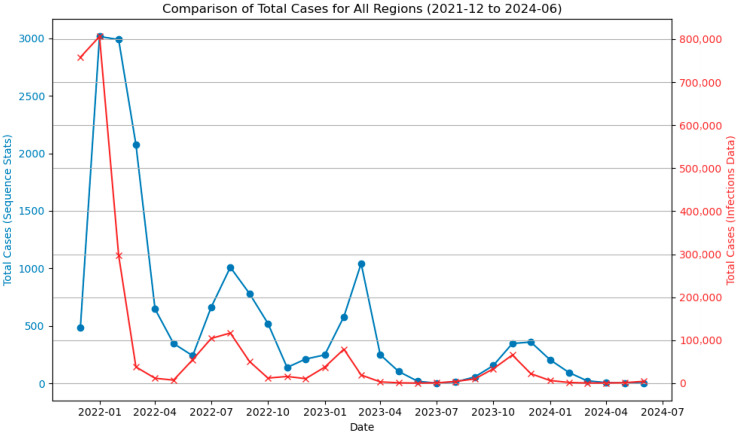
Comparison of total cases for all regions versus data on the number of sequences after subsampling.

The next step was to subsample the sequences, as phylogenetic analysis of such a large number of sequences would require substantial computational resources and time. This also allowed us to address imbalances in sequencing coverage across regions. First, we gathered infection data from the Government Center for Security [8]. We merged daily data from multiple CSV files to create a dataset for the period 1 December 2021 to 30 June 2024. The goal of subsampling was to create a set of sequences that matched both the regional and monthly distribution. This process was implemented using a custom Python v3.8.19 script. The script first loads infection data and calculates the proportion of cases in each region per month. For each region, it reads the corresponding sequence metadata and randomly selects sequences for each month proportionate to that region’s share of national infections. A minimum of 50 sequences per month is enforced to ensure representation. This ensures that the resulting dataset reflects the cross-country temporal and regional distribution of cases. The subsampled regional sequence sets were merged for further analysis. Figure 1 shows the comparison between the number of subsampled sequences and the total number of infection cases.

### 2.3. Filtering Omicron Sequences

We used only Omicron variants with Pango lineages [17] BA.1.1.529, BA.2.*, BA.5.*, XBB.1.*, JN.1.* and CH.1.* for our analysis as they displayed the highest prevalence in Poland during the timeline of the study. We split our dataset into these Omicron groups using a custom Python script. As GISAID uses Pangolin to classify SARS-CoV-2 variants, we used information in the metadata file available in the GISAID download archive—specifically in the “Pango lineage” column.

The initial Polish dataset of investigated Omicron sequences is shown in Table 1.

**Figure 2:**
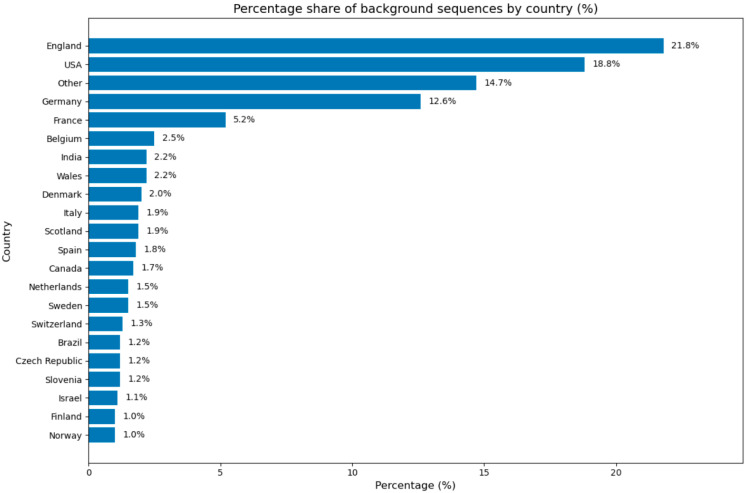
Percentage share of background sequences by country.

After subsampling, a representative background dataset was constructed from Polish Omicron sequence datasets using a similarity-based sequence selection strategy. Precomputed sequence similarity search results were obtained using the AudacityInstant [18] tool (version 5.1.0), integrated with the GISAID EpiCoV™ database (Global Initiative on Sharing All Influenza Data, https://www.gisaid.org, accessed on 1 March 2026), which provides curated global SARS-CoV-2 sequence data. AudacityInstant produces optimized similarity search outputs, including alignment metrics such as bit score, percentage identity, and alignment length. The resulting similarity search data were processed using a custom Python script. Alignments were filtered to retain only those with a minimum percentage identity of ≥99% and covering ≥95% of the query sequence length. For each query sequence, the five top-ranked unique background sequences were selected based on descending bit score. Subsequently, all results were combined, and sequences of Polish origin, as well as duplicate sequences, were excluded from the background dataset. A balanced and diverse background dataset was constructed, representing global SARS-CoV-2 genetic diversity and maintaining close genetic proximity to the analyzed query sequences. The counts of subsampled and background sequences are shown in Table 2. The country of origin of the sequences, broken down by country, is shown in Figure 2.

Finally, we aligned the sequences separately for each variant using MAFFT v7.526 [19] for further analysis and masked them using the tool developed by Nicola De Maio et al. (2020) [20]. We adopted this approach to enhance the reliability of the alignments.

### 2.4. Time-Scaled Phylogenetic Tree Construction

We followed the workflow outlined by Dellicour et al. (2023) [12] to perform a phylogeographic analysis of virus spread. However, we created a Python version of the workflow to address the specific needs of our analysis. We constructed multiple time-calibrated phylogenetic trees using the merged Polish and background Omicron variant sequence datasets. Using IQ-TREE v2.2.03 [21], we generated maximum-likelihood trees based on the GTR model with empirical base frequencies and four gamma rate categories, as selected by ModelFinder. Outlier sequences were identified using TempEst v1.5.3 [22], and after removing outliers, the datasets were reduced by 264 sequences for BA.2.

Time calibration was performed using TreeTime v0.11.4, with a fixed molecular clock rate of 8 × 10^−4^ substitutions per site per year and a clock filter of 4. Sequences deviating significantly from the expected root-to-tip distances were excluded.

### 2.5. Preliminary Discrete Phylogeographic Analysis

We used a discrete trait diffusion model implemented in BEAST v1.10.4 to study the independent introduction events into Poland. Using the previously time-scaled phylogenies as fixed empirical trees, we identified multiple ancestral origins, where the tips changed from any foreign country to Poland.

To examine the geographic origins of Polish SARS-CoV-2 sequences, a discrete phylogeographic framework was applied using BEAST v1.10.4. Time-calibrated phylogenies previously inferred for each lineage served as empirical trees, and only two discrete location states were defined: “Poland” and “other”. Bayesian phylogeographic reconstruction was performed using a Markov chain Monte Carlo (MCMC) approach, with each run conducted independently for one million generations and sampling every 1000 steps. Chain convergence and parameter mixing were evaluated in Tracer v1.7, ensuring effective sample sizes (ESS) of 200 or greater for all model parameters. The first 10% of samples were discarded as burn-in, and maximum clade credibility (MCC) trees were summarized using TreeAnnotator v1.10. Putative introduction events were inferred by inspecting location transitions along branches of the MCC trees. A lineage was considered introduced into Poland if a given internal node was assigned to “Poland”, while its immediate ancestral node carried the state “other”.

### 2.6. Detailed Phylogeographic Analysis of the Regions

We extended our discrete phylogeographic analysis to examine viral transmission within the Polish voivodeships. Each transmission cluster, defined as a time-scaled subtree with at least three sequences from two distinct regions, was analyzed independently.

Sampling locations were treated as discrete traits, and a continuous-time Markov chain model with asymmetric transition rates was applied. The Bayesian Stochastic Search Variable Selection (BSSVS) method identified significant viral transitions between regions. MCMC chains were run for 500,000,000 iterations with sampling every 50,000 steps. Results were evaluated in Tracer, and after removing 10% of samples as burn-in, MCC trees were generated.

To evaluate the statistical support for transitions between specific voivodeships, we calculated adjusted Bayes factors (BFs), which accounted for differences in sample sizes across locations [23,24].

## 3. Results

**Figure 3:**
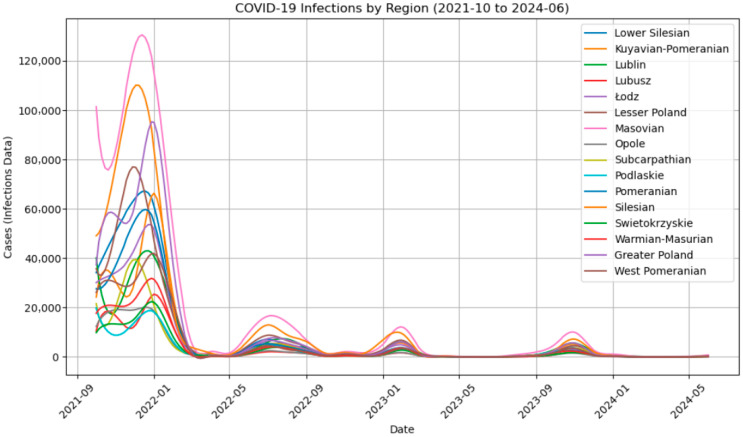
Timeline of infection cases by region.

Infection case reports for all Polish voivodeships at the time of Omicron’s emergence show one large peak of infections in late 2021 and early 2022 and three smaller peaks in Q3 2022, Q1 2023 and late 2023. The regions with the highest number of reported infections between 2021 and 2022 were: the Mazovian, Silesian and Greater Poland voivodeships (Figure 3).

Detailed patient demographic statistics, including age and gender distributions derived from the sequence metadata, are available in the Supplementary Materials (Supplementary Figures S1 and S2).

### 3.1. Introduction Events and Cluster Analysis

To ensure methodological transparency, a cluster attrition cascade was analyzed. Our model initially inferred a total of 5476 introduction events across all lineages (2078 for BA.1, 1289 for BA.2, 1681 for BA.5, 302 for CH.1, 70 for XBB.1, and 56 for JN.1) when no minimum sequence threshold was applied. However, the vast majority of these introductions were singletons or doubletons. Such small clusters typically represent transient, dead-end transmission chains or isolated travel-related cases rather than sustained community spread. To focus explicitly on established, inter-regional transmission networks, we applied a strict threshold requiring clusters to contain at least three sequences from at least two distinct Polish voivodeships. The use of a minimum three-sequence threshold is an established standard in phylodynamic modeling used to eliminate sampling artifacts and ensure that the analyzed clades represent authentic, sustained local transmission (Dellicour et al. [23]).

**Figure 4:**
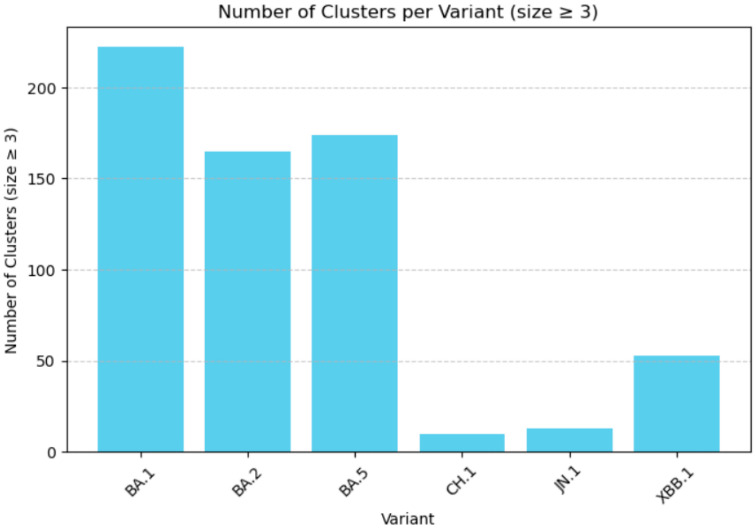
Number of clusters with size ≥3.

Applying this threshold filtered the dataset down to 637 epidemiologically meaningful clusters. The most introductions were observed for the BA.* variant groups. In total, 222 (BA.1), 165 (BA.2), 174 (BA.5), 53 (XBB.1), 13 (JN.1) and 10 (CH.1) introductions generated clusters with at least three sequences, indicating epidemiologically meaningful onward transmission, while all remaining variants together accounted for 23 such clusters (Figure 4).

**Figure 5:**
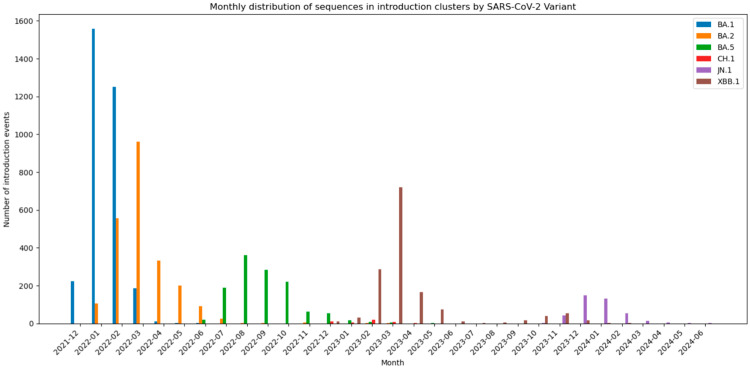
Distribution of sequences in introduction clusters by month.

We also illustrated the distribution of variants in introduction clusters by month, revealing temporal patterns in their spread (Figure 5). We can see that the BA.1 variant emerged in December 2021 with 222 detected sequences, rapidly rising to a peak of 1.557 sequences in January 2022, indicating a swift initial spread. This was followed by a transition to BA.2, which overtook BA.1 in March 2022, peaking at 962 sequences. BA.5 appeared shortly thereafter, dominating by mid-2022, while subsequent variants such as CH.1, JN.1, and XBB.1 displayed staggered but less intense waves of cases starting in late 2022 and into 2023. These data illustrate an almost serial replacement of dominant Omicron subvariants and highlight the dynamic and ongoing evolution of SARS-CoV-2.

To capture each variant’s sharpest epidemic peak, we calculated dominance windows using the central 75% of sequences—thereby focusing on the period when sampling was most intense and excluding both early introductions and long, low-level tails (Table 3).

We can observe that BA.1 remains visible for only two months (January–February 2022) before BA.2 emerges. BA.2 then dominates for roughly four months, through May 2022, after which BA.5 holds the foreground from July to October 2022. The pattern continues with CH.1 circulating at high intensity from December 2022 to March 2023, followed by XBB.1 from February to May 2023 and, finally, JN.1 from December 2023 to February 2024.

**Figure 6:**
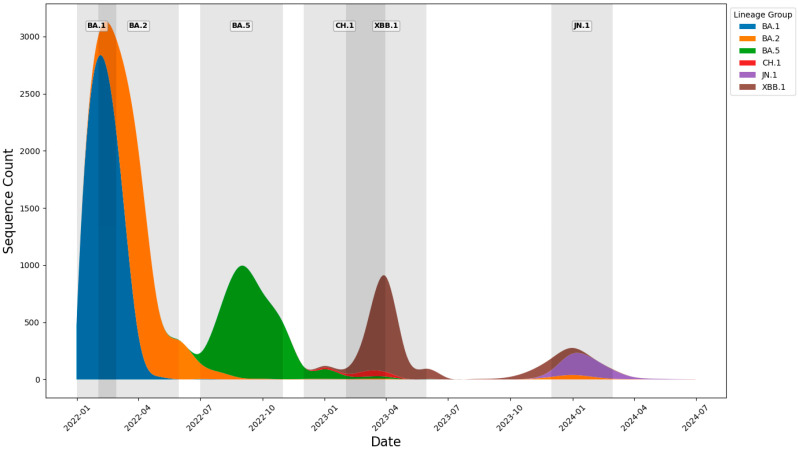
Monthly distribution of investigated Pango lineages with 75% central circulation windows represented by grey area.

To illustrate this on a graph, we overlaid windows representing the central 75% of each Omicron variant’s domestic circulation onto the temporal distribution plot of Pango lineages. These shaded windows shown in Figure 6 align well with the peaks observed in the chart, confirming the consistency between the genomic data and the defined periods of variant dominance.

**Figure 7:**
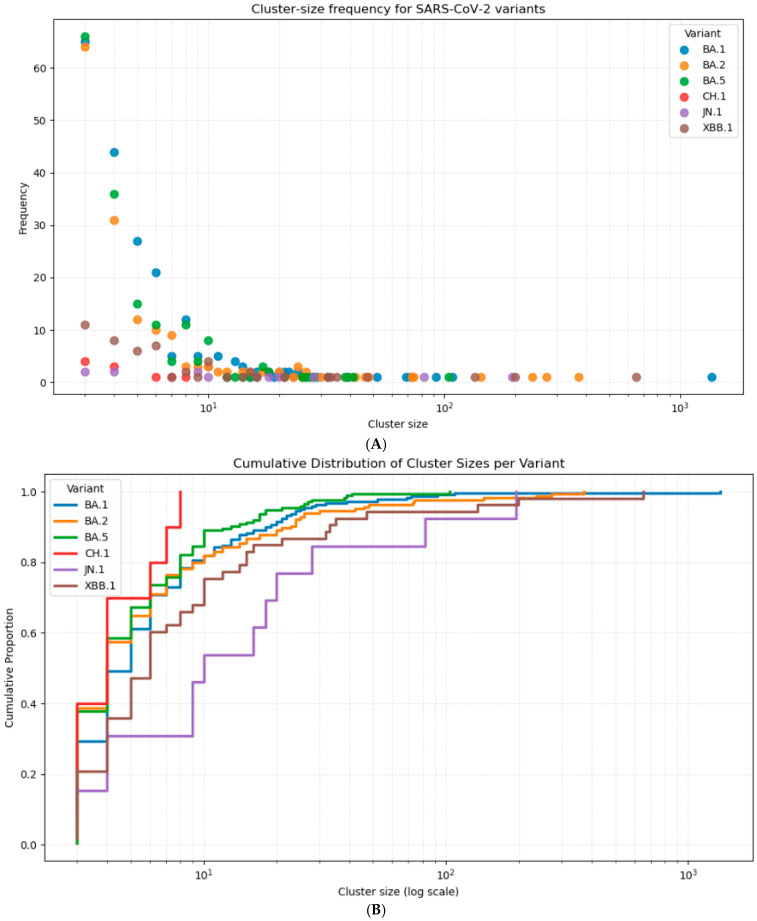
(**A**) Cluster frequencies by size and variant. (**B**) Cumulative distribution of cluster sizes per variant.

Finally, we analyzed the frequency of clusters by size and variant, providing deeper insight into how cluster sizes varied across different variants (Figure 7). To facilitate a clearer comparison between the highly skewed distributions of each lineage, the cumulative proportion of cluster sizes was also visualized. The sizes of top 10 clusters are also shown on Table 4.

### 3.2. Omicron Circulation Between Polish Regions

**Figure 8:**
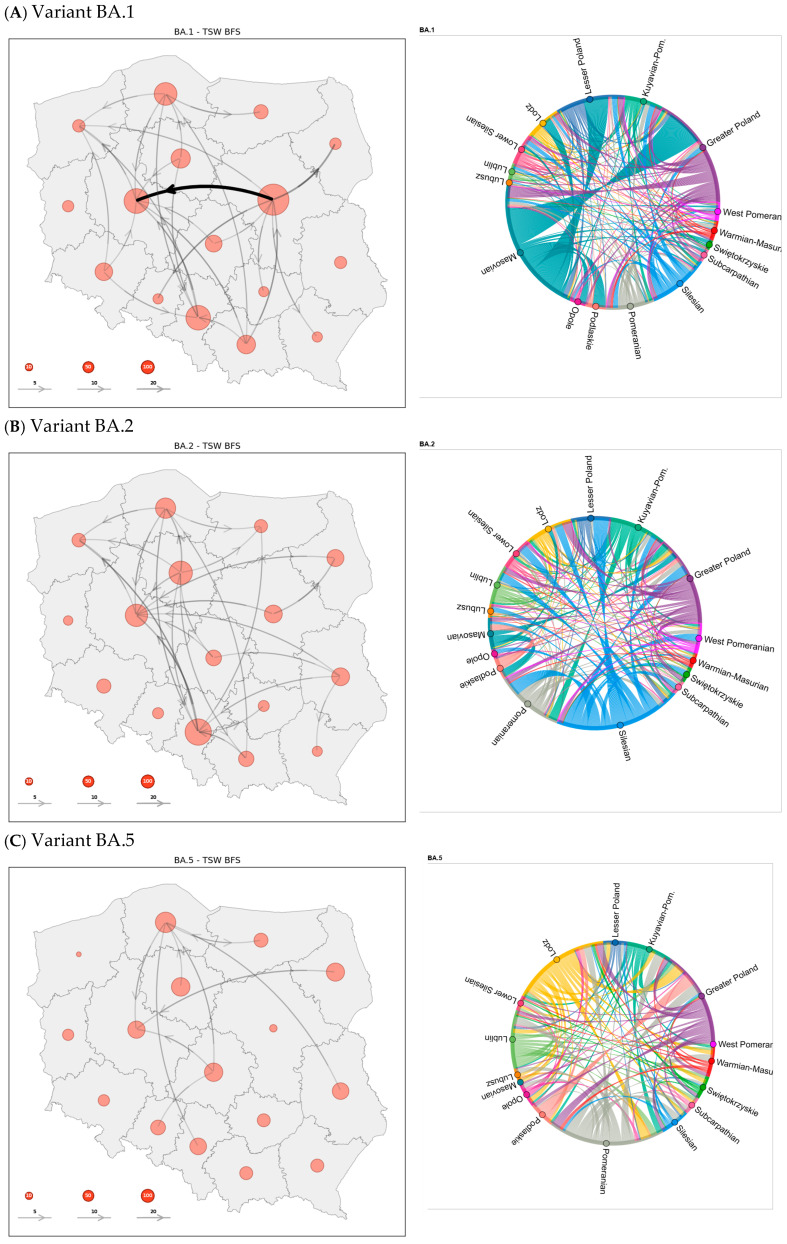

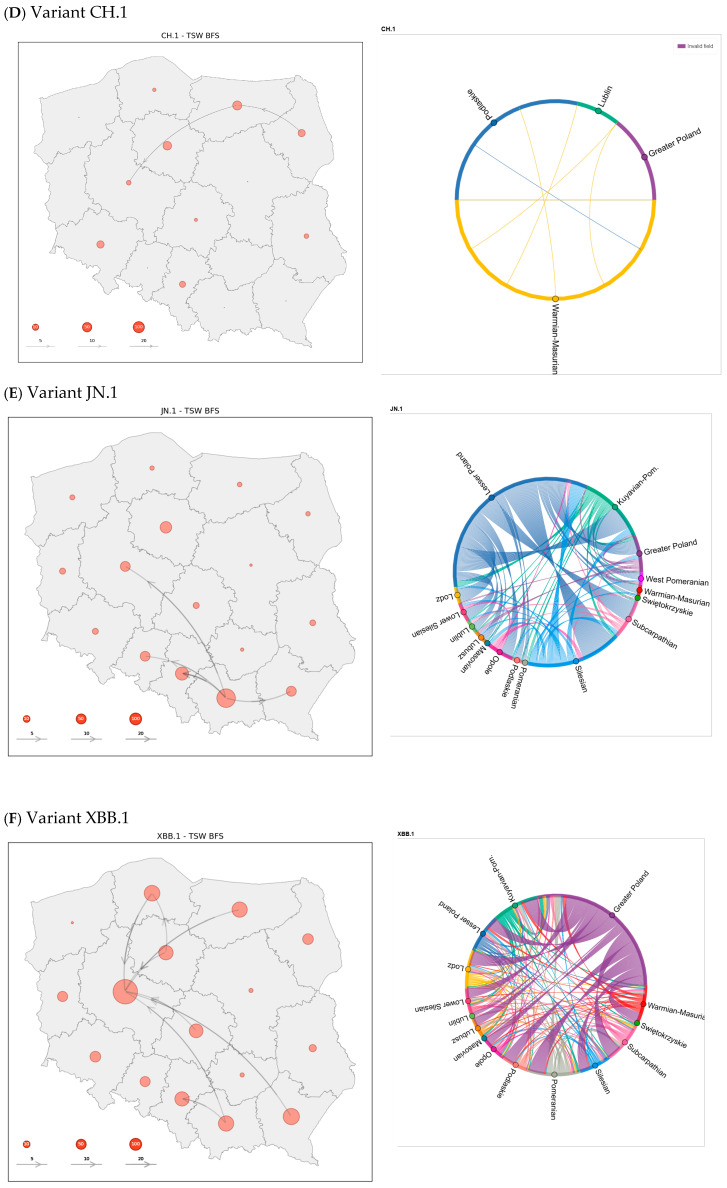
Numeric reconstruction of SARS-CoV-2 lineage movements (**A**–**F**) among Poland’s 16 voivodeships. In our discrete trait analysis, we quantify spatial transitions by reporting the mean frequency of inter-voivodeship dispersal events (indicated by arrows) and intra-voivodeship lineage persistence (represented by transparent red circles), averaged across the posterior distribution of 637 trees. For visual clarity, only transition pathways exceeding 3% of the maximum observed flow are shown. The chord diagram visualizes the inferred lineage dispersal events of the variants between Polish voivodeships. The thickness of the connecting arcs represents the average number of transition events (inter-voivodeship dispersals) calculated across the posterior distribution. The colors of the arcs correspond to the region of origin.

To reconstruct dispersal histories, we generated source-sink maps and circular flow plots (Figure 8A–F) from the discrete phylogeographic analysis for each Omicron lineage. Both visualizations summarize inter-regional SARS-CoV-2 lineage movements among Polish voivodeships.

The analysis of both cluster data and geographical spread shows a major outbreak of the BA.1 variant in the early stage of the Omicron epidemic. During this wave (December 2021–March 2022), export activity was heavily concentrated in the center of the country. The Mazovian province alone generated about 36.1% of all outward movements, while Greater Poland and Silesia contributed 16.4% and 13.7%, respectively. Lesser Poland and Pomerania provided smaller but still appreciable shares, so that five regions together accounted for more than 80% of BA.1 dissemination.

By contrast, the BA.2 period (February–May 2022) was dominated by the industrial south-west: Silesia supplied 33.4% of exports. The main secondary hubs shifted north-west, with Greater Poland (14.9%), the Kuyavian-Pomeranian (10.9%) and Pomerania (9.6%) surpassing Mazovia, whose contribution dropped to only 5.6%. This redistribution signals a marked south-to-north realignment of transmission routes.

Exports during the BA.5 wave (July–October 2022) were more evenly spread but tilted toward the Baltic-central corridor. Pomerania led with 22.2%, followed by Lodz voivodeship (18.5%). Greater Poland (14.2%) and Lublin (10.5%) formed the next tier, while other regions each accounted for less than 8%. No single province dominated as decisively as in earlier waves, reflecting a broader geographic base of spread.

A distinct shift to north-eastern Poland occurred in the CH.1 interval (December 2022–March 2023). Warmian-Masurian alone produced 42.0% of exports and neighboring Podlaskie 24.5%, together driving two-thirds of all outward flow. Lubelskie and Lower Silesia each added roughly 9.0% and 8.6%, respectively, whereas historically important hubs such as Mazovia played virtually no role.

The XBB.1 recombinant (February–May 2023) recentered the epidemic in the west-central regions of Poland: Greater Poland was responsible for 54.1% of exports. Smaller streams originated from Pomerania (8.5%), Kuyavian-Pomeranian (6.0%) and Warmian-Masurian (6.0%), indicating spill-over toward the Baltic coast but little activity elsewhere.

Finally, the brief JN.1 wave (December 2023–February 2024) was driven overwhelmingly by Lesser Poland, which generated 58.0% of all migration events. Silesia (15.6%) and Kuyavian-Pomeranian (10.0%) formed the only substantial secondary sources, leaving the rest of the country with marginal shares.

## 4. Discussion

Our phylogeographic reconstruction, based on 90,136 SARS-CoV-2 sequences from Poland, reveals highly dynamic and regionally shifting circulation patterns of Omicron lineages between 2022 and 2024. The large scale of this genomic surveillance effort allowed us to trace successive realignments of viral dispersal routes. These results demonstrate that the spread of Omicron variants was not static but continuously reorganized in space and time, reflecting a complex interplay of regional connectivity, population mobility, and shifting epidemiological pressures.

The largest clusters (*n* > 100) were identified for variants BA.1, BA.2, BA.5, XBB.1 and JN.1. The dominant cluster corresponds to lineage BA.1, comprising 1.358 sequences, significantly larger than any other group identified. The second- and third-largest clusters belong to XBB.1 and BA.2, with 651 and 371 sequences, respectively. Among the ten contributed Omicron-related clusters, BA.2 recurred with clusters comprising 272, 236, and 143 sequences; XBB.1 recurred with clusters of 199 and 135 sequences; a JN.1 cluster contained 194 sequences. Overall, BA.1, BA.2, and XBB.1 accounted for most large clusters observed during the study period.

A key finding is the distinct geographic signature of each major Omicron wave. The initial BA.1 wave was dominated by the Mazovian voivodeship, the country’s most populous region and home to its largest international airport, suggesting that Warsaw—the capital city of Poland—likely served as the primary entry and dissemination point for this variant.

This pattern shifted dramatically with the rise in BA.2, which was centered in the industrial Silesian region in the south-west, a densely populated area with strong economic ties to neighboring countries, potentially reflecting a different introduction or transmission dynamic. However, earlier studies noted that while Silesia acted as an early gateway for the BA.1 and BA.5 lineages due to its cross-border labor market, the BA.2 variant appeared with a more uniform frequency across all of Poland [25].

Subsequent waves showed further fluidity: BA.5 spread from a broader geographic base along the Baltic-central corridor, CH.1 was concentrated in the more rural north-east, XBB.1 re-emerged in the west-central heartland of Greater Poland and JN.1 was overwhelmingly driven by Lesser Poland in the south. This continuous reorganization highlights that no single region served as a persistent epicenter for the entire Omicron epidemic. Instead, different voivodeships became transient hubs, likely influenced by a combination of factors including regional differences in immunity and local public health responses.

These patterns of long-distance “jump” events followed by localized transmission chains are consistent with findings from other national contexts [13,26], underscoring the universal influence of human mobility and population density in driving pathogen flow.

The dominance windows calculated for the variants with 2- to 4-month intervals show that the period of sustained, high-level transmission for each sub-lineage is short, even though complete “tails” of sporadic detections can stretch much longer in routine surveillance. Successive windows overlap just enough for a smooth hand-off, as each incoming lineage starts its main phase while the preceding one fades, suggesting replacement rather than prolonged co-circulation. The rapid, sequential replacement of variants, with dominant circulation windows lasting only 2 to 4 months, also aligns with global trends of viral evolution under immune pressure. However, the specific regional drivers observed in Poland offer a unique national perspective. The introduction mentioned the significant influx of refugees from Ukraine in 2022, a population with lower vaccination coverage and a significant wave of BA.5 infections [27]. While our analysis did not explicitly track introductions from Ukraine due to data limitations, the shifting epicenters—particularly the emergence of hubs in eastern regions like Lublin during the BA.5 wave—lead to the contextual hypothesis that this demographic event could have contributed to the complex transmission landscape observed. We emphasize that this remains a speculative interpretation rather than a causal mechanism inferred directly from our phylogeographic analysis [28].

In line with similar studies cited earlier, a pronounced challenge remains the potential mismatch between pathogen phylogeny and actual transmission networks, particularly when under-sampling biases exist or when sequencing is sparse in particular geographic areas. Despite considerable advances in sequencing capacity, uneven sampling can obscure smaller-scale or short-lived transmission events. In our study, we addressed this issue by employing geographic subsampling and incorporating chronological information on sample collection. Nonetheless, careful consideration of sampling design—ensuring representation across regions and time points—remains important for accurately inferring transmission pathways.

Furthermore, interpretation of estimated migration events in discrete phylogeographic analyses must be approached with caution. While these models typically perform well in identifying broad directions and intensities of viral flow, they do not always capture immediate epidemiological triggers or highly localized outbreaks. Thus, integrating contact-tracing data or additional metadata (e.g., travel history) can offer a more fine-grained picture of how specific clusters spread in diverse population segments.

## 5. Conclusions

Our phylogeographic analysis of 90,136 Polish SARS-CoV-2 sequences reveals that Omicron circulation was characterized by continuously shifting viral dispersal routes. Rather than relying on a single persistent national hub, successive epidemic waves were driven by transient, regional epicenters governed by local connectivity and epidemiology. This highlights continuously shifting viral dispersal routes driven by regional connectivity and epidemiology, emphasizing dense genomic surveillance value. The operational utility of this integrated approach lies in empowering public health authorities to shift from retroactive observation to proactive, regional decision-making. By identifying emergent, localized epicenters in real-time, policymakers can deploy targeted healthcare resources, initiate localized testing surges, and implement region-specific non-pharmaceutical interventions. Ultimately, this data-driven framework demonstrates how subnational genomic epidemiology can successfully complement centrally mandated measures to mitigate future viral outbreaks.

## Figures and Tables

**Table 1:** Number of investigated Omicron sequences in Polish dataset.

Variant	Polish Sequences
BA.1	25,245
BA.2	7789
BA.5	3440
XBB.1	1733
JN.1	454
CH.1	118

**Table 2:** Number of subsampled Omicron sequences in Poland and number of background sequences.

Variant	Polish Sequences	Background Sequences
BA.1	5515	7930
BA.2	3672	5248
BA.5	3041	4698
XBB.1	1821	2115
JN.1	480	753
CH.1	129	284

**Table 3:** SARS-CoV-2 variant dominance windows.

Variant	Main Circulation Window	Duration
BA.1	Jan–Feb 2022	2 mo
BA.2	Feb–May 2022	4 mo
BA.5	Jul–Oct 2022	4 mo
CH.1	Dec 2022–Mar 2023	4 mo
XBB.1	Feb–May 2023	4 mo
JN.1	Dec 2023–Feb 2024	3 mo

**Table 4:** Top 10 clusters by size.

Variant	No of Sequences
BA.1	1358
XBB.1	651
BA.2	371
BA.2	272
BA.2	236
XBB.1	199
JN.1	194
BA.2	143
XBB.1	135
BA.1	108

## Data Availability

The original data presented in the study are openly available in the GISAID EpiCoV™ database.

## Supplementary Materials

The following supporting information can be downloaded at: https://www.mdpi.com/article/10.3390/v18050520/s1, Figure S1: Distribution of gender; Figure S2: Distribution of patient age.
