# Supplementary material for: Shifting Epicenters: The Dynamic Regional Dispersal of SARS-CoV-2 Omicron in Poland
Source: Viruses. 2026 Apr 30;18(5):520. doi: 10.3390/v18050520 (PMC13211461; doi:10.3390/v18050520)
Supplement: Supplementary file 1 [file viruses-18-00520-s001.zip › viruses-4238872-supplementary.pdf]

Supplementary Materials

Patient Demographics

Analysis of the metadata associated with the subsampled Omicron variant sequences provided the demographic characteristics of the investigated dataset. The gender distribution was relatively balanced, comprising 53.3% (n = 8459) females and 45.7% (n = 7247) males (Supplementary Figure S1). Regarding age distribution, the most frequently observed cohort for both genders was the 30–40 years age group, which accounted for 16.8% of all recorded cases (Supplementary Figure S2).

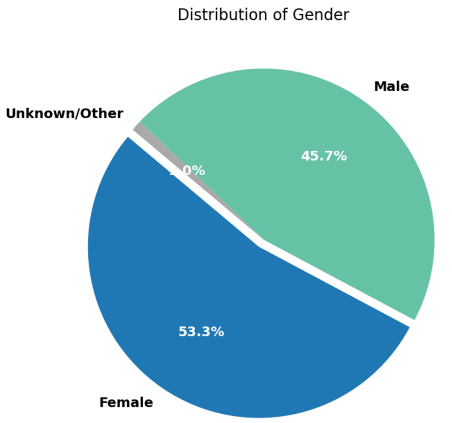

Figure S1. Distribution of gender.

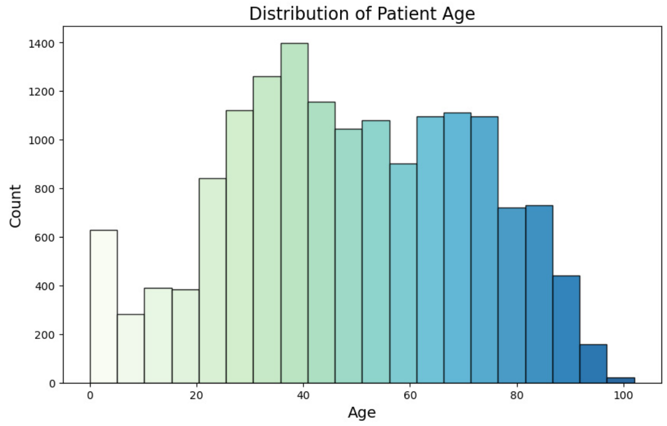

Figure S2. Distribution of patient age.
